# The Glomerulus According to the Mesangium

**DOI:** 10.3389/fmed.2021.740527

**Published:** 2022-01-26

**Authors:** Kerstin Ebefors, Lovisa Bergwall, Jenny Nyström

**Affiliations:** Department of Physiology, Institute of Neuroscience and Physiology, Sahlgrenska Academy, University of Gothenburg, Gothenburg, Sweden

**Keywords:** glomerulus, mesangial cells, crosstalk, glomerular barrier, glomerular diseases, IgAN, DKD

## Abstract

The glomerulus is the functional unit for filtration of blood and formation of primary urine. This intricate structure is composed of the endothelium with its glycocalyx facing the blood, the glomerular basement membrane and the podocytes facing the urinary space of Bowman's capsule. The mesangial cells are the central hub connecting and supporting all these structures. The components as a unit ensure a high permselectivity hindering large plasma proteins from passing into the urine while readily filtering water and small solutes. There has been a long-standing interest and discussion regarding the functional contribution of the different cellular components but the mesangial cells have been somewhat overlooked in this context. The mesangium is situated in close proximity to all other cellular components of the glomerulus and should be considered important in pathophysiological events leading to glomerular disease. This review will highlight the role of the mesangium in both glomerular function and intra-glomerular crosstalk. It also aims to explain the role of the mesangium as a central component involved in disease onset and progression as well as signaling to maintain the functions of other glomerular cells to uphold permselectivity and glomerular health.

## Introduction

The glomerulus is made up of three cell types, the endothelial cells, the podocytes and the mesangial cells (MCs). All three cell types are necessary and dependent on each other for normal glomerular function. During the last two decades, attention has been on the functional properties of the podocytes and to some extent to the contribution of endothelial cells and their glycocalyx to glomerular function and the role of MCs has been less in focus. Herein, the current state of knowledge about the MCs and the mesangium will be reviewed and integrated with recent information about this important cell type having a central role in the glomerulus.

## Location of the Mesangial Cells in the Glomerulus

The MCs make up about 30–40% of the glomerular cell population (1) and are situated between the capillary loops embedded in the mesangial matrix. The glomerular cells, including the MCs, originate from the metanephric mesenchyme during development. The S-shaped bodies organize the endothelium and the podocytes and their respective precursors migrate into the S-shaped bodies while associated stromal mesenchymal cells form the mesangium (2). It has been suggested that Platelet Derived Growth Factor Subunit B (PDGFB) secreted by the endothelial progenitors recruit the mesangial progenitor cells to migrate into the cleft where they promote glomerular tuft formation (3). In the mature glomerulus, the MCs are in direct contact with the endothelial cells but separated from the podocytes by the basement membrane. The MCs are connected to the basement membrane at the paramesangial angles (4). The MCs are also in continuity with the extraglomerular mesangium and the juxtaglomerular apparatus. The MCs are not considered as being a direct part of the filtration barrier but are rather forming a central stalk of the glomerulus where they are important contributors to glomerular function.

## Overview of the Role of The Mesangial Cells in the Glomerulus

**Figure 1:**
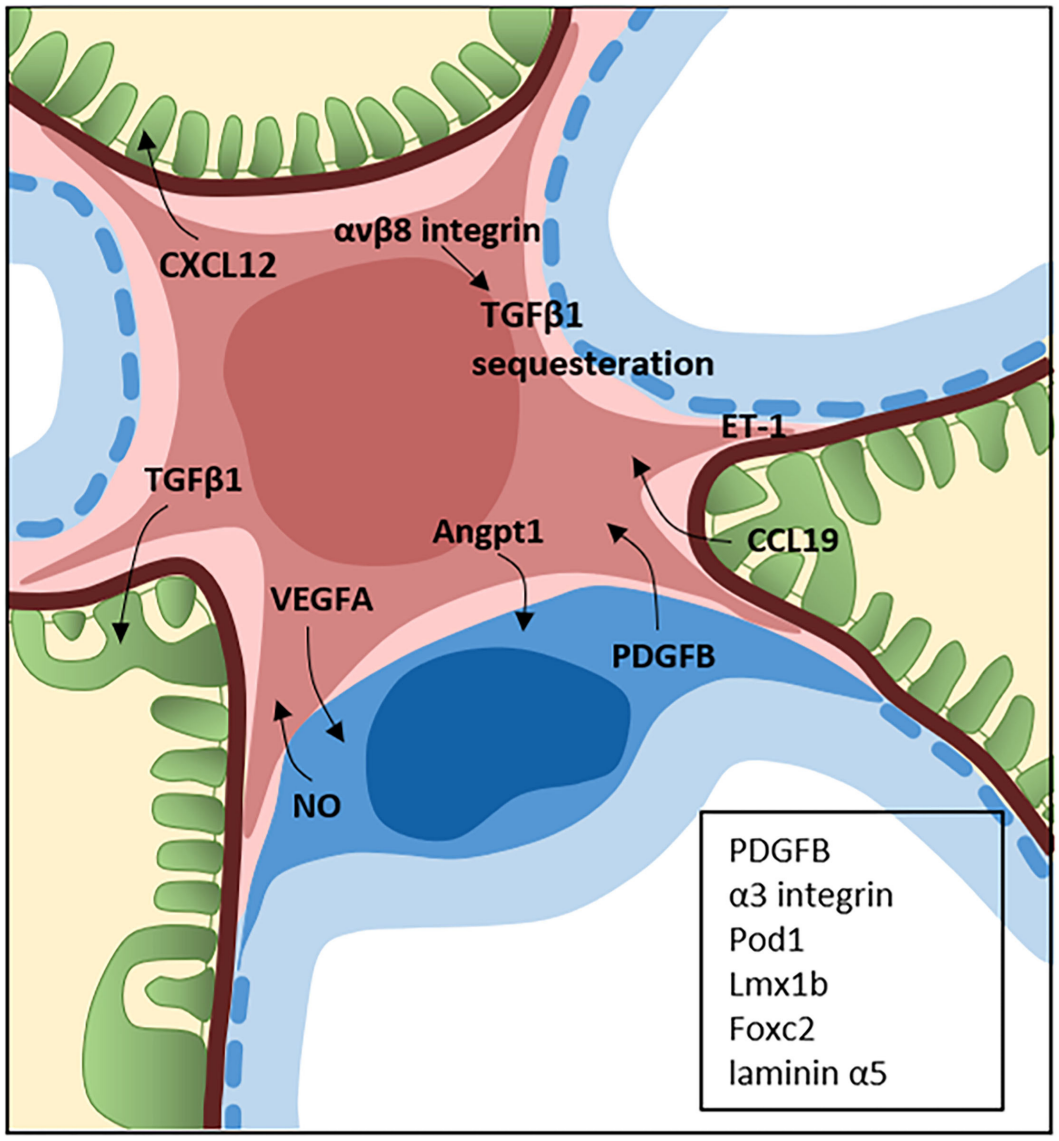
Crosstalk between the mesangial cells, podocytes and glomerular endothelial cells. The central position of mesangial cells (pink) in the glomerulus is a perfect location for crosstalk with both the podocytes (green) and endothelial cells (blue). The field of glomerular crosstalk is emerging and the figure summarizes some of the known crosstalk signaling molecules between mesangial cells and the podocytes and endothelial cells in the normal and diseased state. Endothelin-1 (ET-1) is expressed by all glomerular cells and the crosstalk can occur in several directions. The proteins in the black box represents proteins expressed by the endothelial cells (PDGFB) and podocytes (PDGF, α3 integrin, Pod1, Lmx1b, Foxc2, and laminin α5) which are known to be necessary for normal development of the mesangium.

The MCs have multiple functions such as regulating the capillary surface filtration area, being a source of growth factors and cytokines and clearing the mesangial region from macromolecules entering from the endothelial layer. MCs are considered to be a form of microvascular pericytes with features resembling smooth muscle cells (5). However, the cells have also been shown to possess immune cell-like characteristics such as phagocytic and scavenging properties (6, 7). A recent single-cell transcriptomic study in mice identified the MCs as mesenchymal stromal cells, a class of cells that include fibroblasts, pericytes and vascular smooth muscle cells (8). It has been discussed whether all MCs have similar properties or if there are subclasses of MCs. He et al., using single cell sequencing of both mouse and human glomerular cells, propose that there are distinct subclasses of MCs in the mesangium, including both a prominent pericyte-like MC type and a more fibroblast-like MC type (9). These results also indicate that the MCs possess phagocytic properties as previously suggested (6). Several glomerular diseases affect the MCs such as IgA nephropathy (IgAN), diabetic kidney disease (DKD) and lupus nephritis, to mention a few. However, there is still a lack of knowledge about the exact contribution of the MCs for disease development and especially their role in glomerular crosstalk. The mesangial cells have an important role in clearing the glomerulus of pathogens and deposited extra-glomerular material, in cellular immune responses and in contribution to cell-to-cell signaling in the glomerulus. As the field of crosstalk is emerging, MCs with their location in the glomerulus are highly likely to be central to disease onset and progression (Figure 1).

## Mesangial Cells and the Basement Membrane

The mesangial connection to the glomerular basement membrane has been shown to be of importance for organization of the glomerular capillaries as well as for the contractile properties of MCs. Laminin α5 replacement of laminin α1 in the basement membrane during the capillary loop stage is required for glomerulogenesis. MC adhesion to laminin α5 is mediated by integrin α3β1 and the Lutheran glycoprotein and is necessary for MC organization of the glomerular capillaries (10). More recent work by Zimmerman et al. has shown that nephronectin produced by the podocytes and localized to the glomerular basement membrane may provide an anchoring point for MCs via integrin α8β1. Mice with a conditional deletion of nephronectin in nephron epithelial progenitors or mice with a podocyte specific deletion of nephronectin gave rise to increased numbers of MCs and mesangial matrix and loss of lateral adhesion of the MCs to the glomerular basement membrane (11). Since the mesangial cells are connected to the basement membrane (4) the mesangial cells have been suggested to have a role in regulating the dimension of the glomerular capillaries by changing the extent of the MC-glomerular basement membrane connection (12, 13). Decrease of the capillary lumen has been suggested to occur by pulling the peripheral sites of the glomerular basement membrane centripetally and increase of the capillary lumen by releasing the most peripheral anchoring points of the MCs, thereby regulating the length of the basement membrane (14).

## The Role of the Mesangial Matrix in Normal and Disease Conditions

The MCs form the central stalk of the glomerulus and are embedded in their own self-made mesangial matrix. The generation and turnover of the mesangial matrix is tightly regulated by the MCs themselves but in some glomerular diseases loss of the tight self-control leads to expansion of mesangial matrix and sclerosis. The mesangial matrix is not only important for structural support but is also involved in cell signaling and harboring signaling molecules, for example Transforming Growth Factor Beta 1 (TGFβ1) (15). The mesangial matrix is mainly composed of collagen type IV, collagen type V, laminin, fibronectin and proteoglycans (PGs) (16) but the exact composition of the mesangial matrix is unknown. Agrin and perlecan are two PGs that have been identified in the mesangial matrix (17). Perlecan has been shown to be upregulated in the mesangium in a rat model of chronic transplant dysfunction (17) and in patients with IgAN increased perlecan expression in the glomeruli correlates with slower progression of disease (18). Biglycan and decorin are two PGs that are normally expressed at low levels in the glomeruli but have been shown to be upregulated in renal disease with mesangial expansion resulting in sclerotic areas of the glomeruli (18, 19). Laminin is a major constituent of the mesangial matrix and increased laminin expression in the mesangium has been observed in DKD (20). Nidogens are glycoproteins also expressed in the mesangium in the normal physiological state and are upregulated in the mesangial matrix in patients with glomerular disease (21). Collagen III is normally absent from the glomerulus but has been found in the mesangium in patients with renal disease and correlates with increased mesangial matrix (22). Expansion of the mesangium (increased cell number and matrix) reduce the luminal space and filtration area leading to decreased kidney function. Since a change in the mesangial matrix leading to sclerosis is a major problem in several glomerular diseases, development of drugs targeting this process would be very beneficial in preserving renal function in this large patient group.

## Mesangial Cells Have Contractile Properties

Early studies on the vascular perfusion of amphibian glomeruli led to the observation that blood flow and its distribution between glomeruli and independent capillary loops varied over time (23). These findings suggested the existence of an internal glomerular mechanism for the regulation of blood flow through the glomerular tuft. Subsequent research findings have largely supported this view although some controversy might exist regarding the nature of such regulation. In the 1960's, the first observations were made of the contractile properties in cultured isolated glomeruli (24). The results indicated a role for MCs as source of the contractile force generating the observed glomerular contraction. Later, observations were made showing that vasoactive substances regulating the ultrafiltration coefficient (K_f_), a product of the capillary surface area and hydraulic permeability, also regulated MC contraction (25). These observations led to the suggestion that regulation of blood flow through the glomerular capillaries and regulation of the filtration surface area, was achieved through the contractile properties of MCs (26).

Studies performed both *in vivo* and *in vitro* have demonstrated the contractile properties of whole glomeruli and isolated MCs alike. Micropuncture studies have suggested that mesangial cells are involved in regulating the single nephron glomerular filtration rate (snGFR) by modulating glomerular haemodynamics (27, 28). A more recent study by Ziegler et al. have shown that MCs actively contribute to the regulation of snGFR. They found that MCs contract in response to AngII *in vivo*. By the use of Thy1.1 antibodies to deplete the MCs in rats they showed that the snGFR was reduced and it was no longer affected by Angiotensin II (AngII) stimulation (29).

MCs share certain similarities with smooth muscle cells as they contain a contractile unit consisting of actin and myosin as well as tropomyosin (30, 31). In similarity to the smooth muscle cells, the contraction of MCs is also dependent on an intracellular rise in Ca^2+^, and the phosphorylation of myosin light chains promoting interaction with actin (32). Although evidence points to a role for the MCs as dynamic regulators of glomerular filtration, it should be noted that some findings emphasize that the contractile properties of the MCs mainly serve to stabilize normal glomerular function. Isometric contraction rather than dynamic contraction serves to counteract the forces generated by increased hydrostatic pressures in the glomerular capillaries to maintain glomerular filtration rate (GFR) (31, 33).

## The Regulation of the Contractile Properties of the Mesangial Cells

Contraction of MCs can be initiated by several vasoactive substances. AngII was first shown to cause MC contraction (34), an effect that has been observed repeatedly in both isolated glomeruli and MCs *in vitro*. The binding of AngII to the glomerulus was mainly located to the MC area (35). Arginine vasopressin (AVP) also stimulated MC contraction (34). Endothelin-1 (ET-1), derived from endothelial cells, was further found to cause MC contraction *in vitro* (36, 37). Besides these vasoactive substances, PDGF has also been shown to cause contraction of MCs (38). Conversely, atrial natriuretic peptide (ANP) and nitric oxide (NO) are two substances found to cause relaxation of MCs and isolated glomeruli *in vitro* (35, 39–41).

The importance of Ca^2+^ levels for biological activity and contraction of MCs was demonstrated early (42). Treatment of MCs with vasopressin or AngII altered the intracellular levels of Ca^2+^. Subsequent studies later revealed that MC contraction was a result of the Ca^2+^ initiated activation of plasma membrane chloride channels that in turn generated a depolarization of the plasma membrane and activation of voltage gated Ca^2+^ channels (VOCC). Studies showed that AngII, vasopressin, endothelin and ATP all individually caused a Ca^2+^ mediated increase in chloride conductance (43–45). Further studies showed that the initial Ca^2+^increase was a result of release from intracellular stores (35), later proven to be mediated by the phospholipase C-γ–inositol triphosphate pathway (36, 46–48). The chloride conductance of the plasma membrane was upheld by activation of voltage gated Ca^2+^ channels (49), further increasing intracellular Ca^2+^ concentration and generating contractile forces.

Large calcium activated potassium channels, known as BK_Ca_ channels, are the main regulators of relaxation in MCs. The channel consists of a core α-subunit forming the pore of the channel and one of four accessory β-subunits differently expressed depending on the cell type. In mesangial cells, it is the β_1_-subunit that is expressed (50). When first identified in MCs (51), the BK_Ca_–channel was shown to be activated by Ca^2+^ and membrane depolarization. In the same study, it was shown that the intracellular increase in Ca^2+^ following AngII stimulation could, at least in part, activate the channel. The opening of the BK_Ca_–channel causes a hyperpolarization of the plasma membrane, resulting in a closure of VOCC and inhibition of the chloride and the VOCC positive circuit causing MCs to contract (50, 52). Further studies showed that relaxing hormones such as ANP and NO could activate the BK_Ca_–channel in MCs through the action of cGMP and PKG. These second messengers sensitize the BK_Ca_–channel and decrease its threshold for activation by Ca^2+^ and membrane depolarization (52, 53). Recent research concerning the contraction of MCs has focused on identifying ion channels and possible regulators of contraction, as well as further improving the understanding of the role of MCs in regulation of glomerular filtration. This research has identified a role for the Transient Receptor Potential Cation Channel Subfamily C Member 6 (TRPC6) in MC contraction as well as provided new methods for studying MC contraction *in vivo* in order to establish the role of MCs in regulation of glomerular filtration (29, 54–56).

## Mesangial Cells Can Perform Phagocytosis

MCs can perform receptor independent micro- and macro-pinocytosis and phagocytosis as well as receptor dependent uptake (6). However, some studies suggest that this process is not performed by the MCs *per se*, but rather by a population of cells in the mesangium that have a different phenotype and is responsible for the phagocytic properties of the mesangium (57). The initial report on phagocytosis by MCs was an electron microscopic study where it was observed that MCs could ingest large molecules (58). It was subsequently shown that MCs can take up zymosan particles *in vitro* (59). Thereafter, it was observed that MCs *in vitro* actively take up serum-coated colloidal gold particles via a coated pit mechanism and that the particle ended up in endosomes and phagolysosomes (60). It has also been demonstrated that MCs in culture ingest neutrophils undergoing apoptosis (61, 62). In the anti-Thy1.1 model of mesangial proliferative glomerulonephritis in rats, apoptotic MCs were phagocytosed by healthy neighboring MCs as a mechanism for resolution of hypercellularity (63). Mice deficient in integrin α8 have a delayed healing of glomerulonephritis induced by Habu snake venom compared to wild type mice (64). Using MCs isolated from these mice, it was found that integrin α8 facilitates phagocytosis in MC, likely mediated by integrin α8-cytoskeleton interactions (65). MCs have also been shown to actively take up IgA1. This was determined by incubating MCs with TRITC-labeled IgA1 and after fixation visualizing them with confocal microscopy showing IgA1 in vesicles in the cells. Unfortunately, there was no information on how the IgA1 was taken up by the cells (66). In a recent single-cell RNA sequencing study using human and mouse glomeruli, the MC enriched genes were shown to display several pathways involved in phagocytosis. The results were confirmed *in vitro* by latex bead phagocytosis assays in human MCs as well as *in vitro* by injection of FITC-labeled bovine serum albumin (BSA) in mice showing that the labeled BSA ended up in the MCs (9).

## Mesangial Cells are a Source of Growth Factors and Cytokines

Hyperproliferation of MCs and an increased deposition of mesangial matrix are common occurrences in glomerular disease. Inflammatory processes cause inevitable damage and eventually, as part of the healing process, glomerular sclerosis can ensue causing a decline in glomerular filtration function (67). Recent research has focused on identifying the underlying mechanisms for the above-mentioned events and it has been found that MCs themselves both respond to and secrete various cytokines and growth factors that contribute to these pathological events (68–71).

In the normal state, the MCs are relatively quiescent and secretion of growth factors and cytokines is tightly regulated. However, upon activation by certain stimuli, the MCs will increase their biological activity and secretion. Two of the main actions of these growth factors and cytokines are the initiation of MC proliferation and production and deposition of components of the extracellular matrix (ECM) (72).

TGF-β is a well-known regulator of fibrosis and known to be associated with glomerular disease and the progression of CKD (71, 73). Mesangial cells are known to both act as a target and a source of this important growth factor. In cultured MCs, the expression of TGF-β can, amongst others, be stimulated by mechanical stretch, high glucose, advanced glycation end products (AGEs), AngII, renin, PDGF and platelet activating factor (PAF) (74–78). Some of these factors have also been found to affect the expression of TGF-β receptors, all 3 of which are expressed by MCs (79, 80). *In vitro*, TGF-β has been found to mainly increase the production of ECM components such as fibronectin, collagen I, III and IV as well as proteoglycans (81–84). Concurrently, it has also been found to affect the expression of matrix metalloproteinases (MMPs) as well as increasing the expression of TIMP-2, a tissue inhibitor of MMPs which promotes the deposition of components of the ECM (83, 85). An additional effect of TGF-β in MCs is the induction of expression of PDGF and connective tissue growth factor (CTGF) (75).

CTGF is a growth factor that is implicated in the development of renal fibrosis and DKD (86, 87). MCs *in vitro* are known to upregulate their CTGF-expression in response to stimuli such as high glucose, mechanical strain, AngII, and TGF-β (88, 89). Secretion of CTGF from MCs has further been seen following stimulation with both high glucose and TGF-β for which CTGF acts as a downstream regulator of some of the previously mentioned TGF-β effects such as deposition of fibronectin (75, 90). Similar to TGF-β, CTGF is also known to induce collagen production in cultured MCs (91) and CTGF has also been found to cause MC hypertrophy, a commonly observed occurrence in DKD (92). In addition, mesangial CTGF has been suggested to have a role in enhancing macrophage chemotaxis and adhesion (93).

PDGF is a well-characterized growth factor expressed by MCs and a known stimulator of MC proliferation (70). PDGF is expressed in several different isoforms, A-D, and their receptors consists of dimers of α and β-chains. In MCs, the main receptors expressed are the PDGFR-αβ and PDGFR-ββ. These receptors are primarily activated by binding to dimers of PDGF-B, C and D. MCs are known to express both PDGF-A and B (94–96). Expression and secretion of PDGF from MCs can be stimulated by several factors such as epidermal growth factor (EGF), TGF-β and tumor necrosis factor alfa (TNF-α) as well as PDGF, creating an autocrine loop for growth stimulation (97). Besides proliferation, PDGF can also induce MC migration and production of components of the ECM making the PDGF system an important part of the mechanism underlying mesangioproliferative diseases and renal fibrosis (70).

The inflammatory processes observed in glomerular disease are partly driven by external cells infiltrating the glomerulus as well as by resident cells. MCs are known to secrete cytokines and chemokines that both attract immune cells and affect the MCs themselves (98). The common pro-inflammatory cytokines TNF-α, IL6, IL8, and IL1 are all secreted by MCs and some of these cytokines can also regulate the secretion of cytokines from MCs (99, 100). IL6 was early on shown to be secreted by and to have a mitogenic effect on MCs (101, 102) while having an inhibitory effect on the production of ECM (103). Similarly, IL1 is also known to have mitogenic effects on MCs (100). Besides inducing expression of other cytokines and chemokines, TNF-α is also known to stimulate the expression of CTGF and to regulate cell proliferation and cytotoxicity (104, 105). MCs are known to secrete chemoattractants under experimental settings simulating an inflammatory milieu in the glomerulus. Such chemoattractants are monocyte chemoattractant protein-1 (MCP1), regulated on activation, normal T-cell expressed and secreted (RANTES), IL8 and IP-10 as well as the leukocyte adhesion molecule ICAM-1 (106, 107). The MCs also express chemokine receptors such as CC chemokine receptors type 1 and 7 (CCR1 and CCR7) (106, 108). This suggests that the chemoattractants secreted by MCs are not only serving to attract and recruit leukocytes and monocytes to sites of glomerular inflammation but that the MCs themselves also serve as targets for chemokines secreted during inflammatory processes.

## Mesangial Cells in Glomerular Disease

Several glomerular diseases involve the mesangium either as the entry point of the pathological process or later when the disease progresses. Since an extensive crosstalk is present in the glomerulus between the various glomerular cells, injury to the MCs will eventually lead to damage to the other cells in the glomerulus driving the injury process further with progression of disease ultimately leading to loss of renal function.

In IgAN, the MCs are activated by deposition of immune complexes containing galactose deficient IgA (gd-IgA1). The activation leads to increased production of cytokines, chemokines and complement resulting in MC proliferation and matrix expansion [for detailed review see ref (109)]. Several receptors for immune complexes have been suggested to be located on MCs: the transferrin receptor (CD71) (110), asialoglycoprotein receptor (111), Fc α/μ- (112) or Fc α-receptor (113), α1/β1 and α2/β1 integrin receptors (114) and the β-1,4-GalT1-receptor (115). However, a conclusive result as to a specific receptor is lacking. Recently a paper by Li et al. demonstrated that deletion of microRNA-23b-3p in mice gave rise to IgAN like phenotype with increased mucosal IgA synthesis and IgA depositions in the kidneys along with albuminuria, hypertension and elevated serum creatinine. They propose that microRNA-23b is a potential new therapeutic target for IgAN (116).

DKD is distinctly different from IgAN. It is classified as a microvascular complication of diabetes but eventually the entire glomerulus is affected. In DKD, the first changes seen in the glomerulus are thickening of the glomerular basement membrane followed by mesangial expansion including MC hypertrophy and matrix accumulation leading to sclerosis. Work in the 1970–80s identified that the expansion of the mesangium and the reduction in peripheral capillary surface acts as a constituting mechanism leading to reduced kidney function in DKD (117–120). Accumulating evidence from the last decades suggest that one of the initial pathological events in DKD is a phenotypic transdifferentiation, also known as activation, of mesangial cells into a myofibroblastic phenotype characterized by the expression of α-SMA and production of interstitial collagen. These early pathological cellular changes are associated with the sclerotic events observed in DKD and can be initiated by the common factors driving progression of DKD mentioned below (121–126). Factors that can activate the MCs in DKD include high glucose, dyslipidemia, increased AngII and mechanical stress induced by systemic hypertension. The progression of DKD is mediated by several pro-inflammatory and pro-sclerotic pathways such as the TGF-β and the TNF-α pathways (127). One of the key factors in the sclerotic events in DKD is increased production of TGFβ by the MCs which can be induced by hyperglycemia and AngII leading to increased matrix production by the MCs (128, 129). Another growth factor suggested to be involved in the MC sclerotic process is connective tissue growth factor (CTGF) (130) whose production by the MC is increased by TGFβ stimulation, high glucose and mechanical strain leading to increased matrix production by the MCs (88, 131).

## Mesangial Cells and the Immune System

The general view is that the mesangium has a role in the immune response in many glomerular diseases especially in glomerulonephritis (GN). There is always a question of the importance of factors produced by the cell itself in the onset of disease in relation to factors originating from other cells, tissues or organs. It is clear that the immune system in the most prevalent GN, IgAN, is heavily involved at the level of the B-cells that are known to produce increased amounts of IgA1 and gd-IgA1 (132). The gd-IgA1 has a tendency to form IgA-IgG immune complexes that when escaping clearance by the liver may deposit in the mesangium. It is not likely that the production of gd-IgA1 by the B-cells is the only triggering factor for onset of IgAN since it is known that B-cells may act the same way in healthy individuals without causing disease (109, 133). However, it is generally recognized that the gd-IgA1 immune complexes are a part of the pathogenesis of IgAN (134, 135). The deposits are thought to interact with potential IgA receptors on the surface of MCs triggering an intracellular cascade where cytokines and other pro-inflammatory molecules are released resulting in cellular proliferation and extracellular matrix expansion (136).

## Mesangial Cells and Complement Activation

The complement system is another system that is involved in MC pathology where it is believed to enhance and potentiate injury in glomerular disease. Dysregulation of the complement system is generally observed in many autoimmune disorders and plays a central role in systemic diseases but it is also activated locally in the glomerulus in disease states such as IgAN and DKD. The possibility to block C5 by the use of a monoclonal antibody against C5 in atypical hemolytic uremic syndrome (HUS) has significantly improved clinical outcomes for this patient group (137, 138). Preliminary data on other glomerular diseases are also promising pinpointing the importance of the complement system in glomerular disease onset and progression (139).

Among the three complement pathways, the alternative pathway seems to be the main pathway activated in MCs at least in IgAN. C3 deposits are present in over 90% of IgAN cases often along with properdin and factor H (140). The gd-IgA1 forming complexes have been suggested as triggers of C3 along with IL-6 and proliferation of the mesangial cells (141). Hydrolysis of C3 leads to an increase in C3a and C3b. C3b causes formation of C3 convertase and thereafter C5 convertase. It was previously reported that alternative complement pathway components such as Factor P, Factor B and complement factor H can be detected in kidney tissue in IgAN, and elevated levels of Factors P and B are found in the circulation of patients with IgAN (142). In addition, it is known that the lectin pathway also can be activated by polymeric IgA in MCs in IgAN causing deposition of C4 but this pathway is activated to a lesser extent compared to the alternative pathway. Alternative pathway complement components (Factors B and P, CFB, and CFP) and complement regulatory protein (complement factor H, CFH) are widely present in the kidney tissues of patients with IgAN and there are also significantly increased CFB and CFP levels in the circulation of patients with IgAN. Terminally, C5b-9 [also called membrane attack complex (MAC)] is being formed and deposits of MAC are frequently seen in IgAN (143, 144). The lectin pathway is involved in both IgAN (145) and in IgAN vasculitis through upregulation of C3 acting on MCs (146).

Complement is also involved in the disease progression of the most common cause of end stage renal disease (ESRD); diabetic kidney disease. Less is known about the MCs and their involvement but there is clearly an upregulation of complement in DKD and glycated end products are thought to render glomerular cells prone to complement upregulation. Most studies mention complement upregulation in endothelial cells, podocytes and tubular cells. Of the three possible complement activation pathways, the alternative and the lectin pathways seem more upregulated (147, 148). Less is known about the MCs but C5a is upregulated along with many other complement molecules both systemically and locally in the mesangium (149). It has also been shown that inhibition of C5a could attenuate mesangial proliferation in rats with experimental DKD (150).

## *In Vivo* and *In Vitro* Models for Investigating Mesangial Function in Health and Disease

The most commonly used *in vivo* model for studying mesangial function is the Anti-Thy1.1 model (151) and models of mesangial proliferation such as the Habu snake venom model (152). Administration of anti-thymocyte serum or anti-Thy1.1 antibody to rats causes mesangiolysis with following mesangial proliferation (Anti-Thy1.1 nephritis) and is a model of mesangial proliferative glomerulonephritis (151). Administration of Habu snake venom to rats gives rise to segmental mesangial proliferation (152). As there is no specific protein exclusively expressed by MCs, generation of mice knock out models specifically targeting mesangial genes is not possible. On the other hand, attempts to study MC gene function has been made using the *FoxD1-cre* mouse line (153, 154). FoxD1 is not exclusively expressed by MCs but is expressed by a population of progenitor cells that give rise to renal stroma, pericytes, vascular smooth muscle cells and MCs (155–157).

There are also *in vitro* models that are more specific for glomerular diseases affecting the MCs. For IgAN it has been difficult to establish a good mouse model. This is mainly due to the lack of IgA1 in species other than primates and galactose-deficient IgA is a form of IgA1 lacking sugars in the hinge region. Existing murine models of IgAN are excellently reviewed in detail elsewhere (158). In short, the two most recent murine IgAN models are the grouped ddY mouse and the α1KI-CD89Tg mouse. The grouped ddY mouse was established by inter-crossing early onset ddY mice (159). The ddY mouse strain is a spontaneous IgAN model where the mice develop mesangial IgA depositions with co-deposits of IgG, IgM and C3 (160). The α1KI-CD89Tg mouse expresses both human IgA1 and CD89 resulting in mesangial deposits of IgA1-sCD89 complexes resulting in kidney inflammation, hematuria and proteinuria similar to human IgAN (161).

For DKD, there are different models depending on whether the aim is to recapitulate DKD from type I or II diabetes but most models give rise to mesangial proliferation and mesangial matrix expansion at varying levels. The most commonly used type I diabetes model uses streptozotocin (STZ) as STZ leads to irreversible pancreatic beta cell apoptosis. There are also genetic models of type I diabetes in mice, e.g., the Akita *Ins*2^+/C96Y^ model, but one of the drawbacks of this model is that only male mice develop hyperglycaemia. For type II diabetes (insulin resistance), the db/db or ob/ob has been widely used in combination with high-fat feeding. The problem with most mouse models of DKD is that renal damage is limited, usually takes a long time to establish and only partly recapitulates human disease. More information regarding mouse models of DKD is found in reference (162).

MCs are rather easy to culture from glomeruli obtained from animals and humans. They are usually characterized by the expression of smooth muscle actin, PDGF receptor β and vimentin and negative expression for markers of parietal cells, endothelial cells and podocytes (163). Recently is has been shown that PDGF receptor β is expressed not only by the MCs in the glomeruli and for identifying true MCs a set of genetic markers has been suggested (PDGFRB, PDGFRA, GATA3 and CNN1) (9). MCs can also be cultured from glomeruli obtained from needle biopsies of patients with IgAN (164). It is worthy to note that MCs cultured *in vitro* express smooth muscle actin, a marker that is not usually expressed in the mature healthy glomerulus *in vivo*. MCs expressing smooth muscle actin are considered to be activated and/or dedifferentiated as seen *in vivo* in disease states and have been described as a glomerular myofibroblast (122). If cultured for a longer time period, MCs may form nodular structures and these structures were shown to have less smooth muscle actin and a phenotype more resembling MCs *in vivo* (165).

To recapitulate mesangial disease MCs can be cultured in a diabetic milieu to mimic diabetic conditions or stimulated with gd-IgA1 to mimic IgAN. Cells can also be stimulated with growth factors important for mesangial proliferation and fibrosis such as PDGF and TGFβ or pro-inflammatory mediators like IL-1β. In addition, it is easy to knock down genes of interest in MCs in culture to investigate the role of different proteins for mesangial function. For studies of mesangial crosstalk *in vitro*, the most common setting has been medium transfer or co-culture (please see crosstalk section for examples). New and exciting *in vitro* models for glomerular crosstalk include glomerulus-on-a-chip and organoids but unfortunately the glomerulus-on-a-chip models that have been developed do not include MCs (166) and MCs are so far missing or underrepresented in kidney organoid glomeruli (167–169), possibly due to the lack of vascularization of the organoids.

## The Role of Mesangial Cells in Glomerular Crosstalk

The MCs are in direct contact with glomerular endothelial cells and separated from the podocytes by the basement membrane. Although MC crosstalk is understudied compared to podocyte and endothelial crosstalk, their central position in the glomerulus and close contact to the other cell types supports their role as a central hub in the glomerulus likely to contribute significantly to glomerular crosstalk. For example, angiopoeitin1 is expressed by both podocytes and MCs and the receptor, Tie1, is found on the endothelial cells. In mice with induced deletion of Angpt1 at E10.5, reduced numbers of MCs were observed (170).

## Mesangial-Endothelial Crosstalk

MCs are dependent on PDGF-B from the endothelial cells for their development. This was demonstrated by genetically deleting PDGF-B production in glomerular endothelial cells rendering only a single vascular sack per glomerulus resulting in the death of the mice before birth (171). Knock down of PDGF receptor β gives a similar result with glomeruli lacking MCs and the mice die shortly after birth (172). In a co-incubation experiment of bovine aortic endothelial cells and rat MCs, it was found that stimulating NO release from the endothelial cells with bradykinin caused changes in cGMP in the MCs (173). These findings were also confirmed by others (39). Integrin αvβ8 is expressed by MCs and is known to reduce TGFβ signaling by sequestering it. In mice, deletion of integrin αvβ8 leads to glomerulopathy due to reduced latent TGFβ binding. This leads to increased bioavailability of TGFβ and induction of endothelial cell apoptosis suggesting that MCs impact TGFβ signaling which in turn influences endothelial cell function (174). Endothelial to MC crosstalk has also been shown by transferring exosomes from endothelial cells cultured in high glucose to MCs. The high glucose treated endothelial cells secreted a higher number of exosomes and they were highly enriched in TGF-β1 mRNA compared to cells cultured in normal glucose. The exosomes were taken in by the MCs and promoted cellular proliferation and extra cellular matrix production through the TGFβ1/Smad3 signaling pathway (175).

Co-culture of MCs and human umbilical vein endothelial cells (HuVEC), rendered a lower concentration of endothelin 1 (ET-1) in the cell culture media. It was demonstrated that this was due to down-regulation of endothelin converting enzyme 1 (ECE-1). Losartan abolished the downregulation of ECE-1 in the co-culture and AngII induced inhibition of ECE-1 expression in HuVECs suggesting AngII I can be one of the mediators involved in the ECE-1 down regulation. This shows that the bioactivity of ET-1 is regulated not only by the endothelial cells but also by the surrounding cells demonstrating crosstalk between the cells (176). ET-1 crosstalk between endothelial cells and MCs has also been demonstrated in a study investigating the role of the endothelin B receptor in diabetes. Using the streptozotocin model of diabetes in ETBR^−^/^−^ mice, increased expression of ET-1 was found in these mice compared to controls. *In vitro* experiments showed that conditioned medium from high glucose treated ETBR^−/−^ glomerular endothelial cells promoted MC proliferation and increased matrix related proteins. Similar effects on the MCs were achieved by ET-1 knock out in glomerular endothelial cells or inhibition of ET-1/endothelin A receptor in glomerular endothelial cells (177). Crosstalk has been demonstrated between MCs and endothelial cells in mesangial proliferative glomerulonephritis (MPGN) using the Anti-Thy1 nephritis model and co-culture of MC and endothelial cells. In the anti-Thy1 model, endothelial proliferation can be seen beside mesangial proliferation and the authors investigated the connection. They found that in anti-Thy1 nephritis mesangial cells express VEGFA and the endothelial cells increased their expression of angiopoietin 2 (Angp2). Using a co-culture system, it was confirmed that MCs activated by PDGF-BB expressed VEGFA leading to activation of VEGF receptor 2, Angp2 expression and endothelial cell proliferation. Increased Angp2 inhibited Tie2 phosphorylation and enhancing Tie2 phosphorylation by Vasculotide alleviated endothelial cell proliferation on day 7 of the anti-Thy1 model. This was suggested as a strategy to lessen the vascular lesions in MPGN (178).

## Mesangial-Podocyte Crosstalk

The relationship between podocytes and MCs has been described in the developing kidney where several knock out and mutation experiments have demonstrated that MC recruitment and adhesion is dependent on proteins expressed by the podocytes. Several genes expressed by podocytes (α3 integrin, Pod1, Lmx1b, Foxc2) are needed for proper formation of the glomerular capillary loops and mesangium and mice lacking these genes have defects in MC recruitment, glomerular capillary loops and podocytes (179–183). In addition, laminin α5 in the basement membrane is needed for adhesion of MCs to the glomerular basement membrane via the G domain of laminin α5 and this is crucial for normal glomerular capillary loop development and a normal mesangium (10). Another proof of MC and podocyte crosstalk is that mutations in Wilms tumor suppressor gene gives rise to mesangial sclerosis (184). Another way for podocytes and MCs to communicate is through chemokines. This has been demonstrated by MC expression of the chemokine receptor CCR7 and its ligand expressed by the podocytes (CCL19) and the receptor CXCR4 receptor expressed on podocytes and the ligand (CXCL12) expressed by MCs (108, 185). In DKD, endoplasmic reticulum (ER) stress has been suggested to be part of the disease progress. Culturing MCs in high glucose and transferring the medium to podocytes led to inhibition of the endoplasmic-reticulum-associated protein degradation pathway (ERAD) and podocyte injury. In diabetic mice inhibition of ERAD resulted in increased albuminuria, podocyte apoptosis and reduced nephrin expression (186). The identities of the specific molecules produced by the MCs leading to podocyte damage are unknown.

Podocyte-MCs crosstalk has also been investigated in the setting of IgAN, where such crosstalk is important in driving the glomerular damage seen in IgAN. Podocytes do not bind IgA from patients with IgAN (gd-IgA1) and stimulation of podocytes with gd-IgA1 does not induce release of growth factors or cytokines. However, transferring medium from human MCs stimulated with gd-IgA1 lead to increased expression of TNF-α as well as CTGF and increased expression of the TNF-α receptors on podocytes reducing important podocyte markers and increasing podocyte apoptosis (187–189). Medium transfer from MCs treated with gd-IgA1 induced epithelial-to-mesenchymal transition in podocytes and the PI3-K/Akt pathway was involved in the process (190). gd-IgA1 stimulation of MCs has also been shown to upregulate TGFβ1 and CXCL1. Medium from MCs treated with gd-IgA1 or CXCL1 in combination with TGFβ1 reduced podocyte adhesion and increased podocyte cell death (191). Increased TGFβ1 expression after gd-IgA1 stimulation of MCs *in vitro* has been reported (164, 188) as well as in glomeruli from patients with IgAN (18).

In summary, there is an emerging view that crosstalk between the MCs and the other cells in the glomerulus is active and ongoing during development in the normally functioning glomerulus and during disease.

## Conclusion

In conclusion, over the years the role of the MCs in the glomerulus has been extensively studied and existing data suggest a central, pivotal role for MCs in glomerular function. In some forms of glomerular disease the MCs are heavily involved and are likely to be central for disease onset and progression. There are still some areas, especially concerning the role of MCs in glomerular crosstalk, that are less well-studied both in the normal state and in disease conditions.

## Author Contributions

KE have taken the lead in the final editing of the review. JN has finalized and submitted the review. All authors have planned and written the manuscript and contributed to the review.

## Funding

This work was supported by the Swedish Research Council (2019-01394), the Inga-Britt and Arne Lundberg Research Foundation, the National Kidney Association, and the Sahlgrenska University Hospital grant ALF (965544).

## Conflict of Interest

The authors declare that the research was conducted in the absence of any commercial or financial relationships that could be construed as a potential conflict of interest.

## Publisher's Note

All claims expressed in this article are solely those of the authors and do not necessarily represent those of their affiliated organizations, or those of the publisher, the editors and the reviewers. Any product that may be evaluated in this article, or claim that may be made by its manufacturer, is not guaranteed or endorsed by the publisher.
